# Russian bank data: Birth and death, location, acquisitions, deposit insurance participation, state and foreign ownership

**DOI:** 10.1016/j.dib.2019.104560

**Published:** 2019-10-04

**Authors:** Alexei Karas, Andrei Vernikov

**Affiliations:** a Utrecht University, University College Roosevelt, Lange Noordstraat 1, 4331 CB, Middelburg, the Netherlands; b Utrecht University School of Economics, the Netherlands; c Russian Academy of Sciences, Institute of Economics, Nakhimovsky prospekt 32, 117218, Moscow, Russia

**Keywords:** Russia, Bank data, State-owned bank, Foreign-owned bank

## Abstract

For every Russian bank we hand collect information on its registration, license revocation, liquidation, location changes, acquisitions, entrance to and exit from the Deposit Insurance System as well as state and foreign ownership. The data come from various sources freely available online. Yet, some of these data are hard to find or require some tedious work to organize. This paper brings the information together and makes it publicly available. Combined with Russian bank financial statements (freely available on Bank of Russia's website) this dataset provides ample possibilities for empirical research: on bank failure prediction models; on how acquisitions affect competition; on how bank efficiency varies with ownership; on how a particular event, such as acquisition, change of location or ownership, or acceptance to deposit insurance, affects bank behavior and performance.

**Table:** Specifications Table

Subject	Finance
Specific subject area	Empirical Banking in Russia
Type of data	Table (Excel Format)
How data were acquired	Hand collected from various public online sources
Data format	Raw
Parameters for data collection	Events and attributes for all Russian banks that ever existed
Description of data collection	Hand collected from various public online sources
Data source location	Russia
Data accessibility	Repository name: Mendeley Data
	DOI: https://doi.org/10.17632/vszrz2dpb3.1
	URL: https://data.mendeley.com/datasets/vszrz2dpb3/1

**Table:** 

**Value of the Data** • Much information on Russian banks is (or once was) freely available online. Some of that information, however, is hard to find or requires some tedious work to organize. This paper brings the information together and converts it into a user-friendly format. • The data benefits researchers at universities, central banks and international organizations (such as the World Bank and the IMF) who study banking in Russia or perform cross-country comparisons of various banking systems. • Combined with Russian banks' financial statements, freely available on the Central Bank of Russia's (CBR) website, this dataset provides ample possibilities for empirical research. The dramatic increase in bank license revocations in 2014–2019 provides rich testing ground for various failure prediction models. The large number of acquisitions (especially since 2008) invites questions about their effects on bank efficiency, competition and financial stability. Improved data on state and foreign bank ownership encourage a re-examination of earlier findings on the relative efficiency of Russian banks [1,2]. Finally, the database supports studies of how a particular event, such as acquisition, change of location or ownership, or acceptance to deposit insurance, affects bank behavior and performance. • The data on state and foreign ownership are unique in coverage: the sample period extends from 1991 to 2019. • The data on state and foreign ownership are unique in detail: we sub-divide each ownership category into homogenous sub-groups. The main criteria of this sub-division are the type of the main controlling party and the business model. For some research purposes working with specific sub-groups, rather than the category as a whole, may be preferable to avoid the apples-to-oranges comparison.

## 1 Data

The dataset [3] represents a table in Excel. Variables are in columns; variable names are in the first row. Rows correspond to different observational units, that is, different Russian credit institutions. Each institution is identified by its unique official registration number reported in the first column. Credit institutions are of two types: banks and non-banks. Non-banks include settlement and clearing institutions, such as Western Union and PayPal. The type (bank or non-bank) is reported in the second column. In the remaining text we, unless explicitly stated otherwise, do not distinguish between banks and non-banks and, for brevity, refer to all institutions as banks. The remaining columns provide information on various events that happened during the bank's lifetime as well as on various attributes the bank (temporarily) possessed. Table 1 describes the details. We split the table in two panels because the sources used for variables in Panel A differ substantially from the sources used for Panel B.

**Table 1:** Variable description.

Variable	Description
**Panel A**	
regn	Unique registration number assigned to every credit institution by the CBR
nko	Binary variable: 0 = bank, 1 = non-bank
regist	Date when the bank was officially registered by the CBR
revok	Date when the bank lost its banking license
likvid	Date when the bank was formally liquidated (stopped existing)
dienter	Date when the bank was included in the deposit insurance system
diexit	Date when the bank was excluded from the deposit insurance system
reorg	For banks that got acquired: registration number of the acquirer and acquisition date (ddmmyyyy) separated by semi-colon. Example: 2275; 20092005
acq1	For banks that acquired other banks: registration number of the first target and acquisition date (ddmmyyyy) separated by semi-colon. Example: 3310; 28092001
acq2	Same as above for the chronologically second target
acq3, etc.	Same as above for the chronologically third target, etc.
o100f1	First uninterrupted spell of time (ddmmyyyy-ddmmyyyy) during which a bank was listed by the CBR as 100% foreign-owned. Example: 01042005-01042019
o100f2	Second uninterrupted spell
o100f3, etc.	Third …, etc.
address0	Original official location of headquarters: address (in Cyrillic), federal subject, and federal district separated by semi-colons.
address1	After first change of official location: address (in Cyrillic), federal subject, federal district, and date of location change (ddmmyyyy) separated by semi-colons.
address2	After second change of official location: …
address3, etc.	After third …, etc.
**Panel B**	
	Start and end dates (ddmmyyyy) separated by a dash (example: 01072018-01012019) of a period during which the bank was:
o50sFED	controlled by the federal government or the CBR
o50sREG	controlled by the government of a federal subject (region)
o50sMUN	controlled by a municipal authority
o50sSOE	controlled by a non-financial state-owned enterprise
o50sSOB	controlled by another state-owned bank
o50sSCO	controlled by a state corporation
o50fSUB	a subsidiary of a foreign privately-owned bank
o50fGOV	controlled by a foreign state-owned bank or another public sector entity
o50fOTH	another foreign-controlled bank

## 2 Experimental design, materials, and methods

The dataset includes 3176 banks. This list emerged gradually as we worked with our sources: whenever we found a bank not on our list, we added it.

Our primary two sources for Panel A variables of Table 1 are the websites of the Central Bank of Russia (CBR) [4] and the Deposit Insurance Agency (DIA) [5]. When we can't find relevant information on those two websites we consult the Memory Book of Banki.ru [6]. When we find the relevant information we copy-paste or type it over into our database. Below we provide the details.

For existing banks records of *regist* and *nko*-status come from the most recent list of existing credit institutions published by the CBR [7]; records of *revok* come from the most recent list of revoked licenses published by the CBR [8]. For banks liquidated after Jan 1, 1999 records of *regist*, *revok*, *likvid*, and *nko*-status come from the quarterly lists of liquidated banks published by the CBR [7]. For banks liquidated before Jan 1, 1999 records of *regist*, *likvid*, and *nko*-status come from Ref. [9]; records of *revok* come from the Memory Book of Banki.ru [6].

We take records of *dienter* and *diexit* from the most recent list of banks participating in the Deposit Insurance System published by the DIA [5]. We collect information on acquisitions from the quarterly lists of acquisitions published by the CBR since 1999 [7]; pre-1999 records come from Ref. [6]. We collect information on 100% foreign ownership *o100f* from the quarterly lists of 100% foreign-owned banks published by the CBR since 1999 [7]; pre-1999 records are not available.

We fill in variables *address0, address1, address2, …*for banks that at least once changed their official location. For these banks the CBR reports the date of each location change as well as the bank's address before and after the change. This information is published since Jan 1, 1999 [7]. For all other banks we treat their address as permanent and only fill in their original location *address0*: this information comes from the CBR's most recent list of existing credit institutions [7], similar lists published in the past, or [9]. Next to each address we report the corresponding Russia's federal subject and district. The list of 85 federal subjects comes from Ref. [10]. Federal districts include the following: Central, Far Eastern, North Caucasian, Northwestern, Siberian, Southern, Ural, Volga.

Panel B variables of Table 1 represent an outcome of a multi-year data collection effort. The first findings of this effort were published in English in 2012 [11]. Since then the data were refined and expanded. Below we summarize this process.

We search for ownership information on every bank (but not non-bank) on our list. Specifically, we screen the information disclosure of the bank itself and of its parent entities for the presence of public or foreign entities among bank's shareholders or the shareholders of the bank's shareholders. When available, we use the special disclosure form that lists the controlling parties of each bank published in Ref. [9]. When not available, we consult various other sources including the bank's website, the websites of the bank's key shareholders, as well as [4,6].

Once we find evidence of control by public or foreign entities (we provide the definition of control below) we search for information on how this control changed over time: the date when the bank became controlled by public or foreign entities, the changes in the main controlling parties over time, and the date (if available) when this control stopped. We then put such a bank on our watch list, and closely monitor any changes in its ownership structure over time. Finally, we continuously screen financial news and official reports for potential new candidates to be added to the watch list.

We define a bank as state-controlled if 50% or more of its equity collectively belongs to public entities. We distinguish between the following public entities:

1. FED — federal government or the CBR;
2. REG — government of a federal subject (region);
3. MUN — municipal authority;
4. SOE — non-financial enterprise whose equity is more than 50% owned by (1)–(3) above;
5. SOB — another bank whose equity is more than 50% owned by (1)–(3) above;
6. SCO — state corporation (goskorporaciya), i.e. a statutory corporation established and governed by an individual law (e.g. VEB.RF, Deposit Insurance Agency, Roskosmos, and Federal Corporation for SME Promotion).

Once a bank is defined as state-controlled, we assign it to one of the six categories (*o50sFED, o5*0sREG*, o50sMUN, …*) corresponding to the type of entity that exercises the most control. This assignment is done on a case-by-case basis and involves judgment. To make this judgment we consider all (hard and soft) available information:

• the exact equity shares held by each type of entity;
• their relative representation on the Board of Directors;
• the bank's main clients and business partners, etc.

We define a bank as foreign-controlled if 50% or more of its equity collectively belongs to foreign entities. Screening ownership information of parent entities proves particularly important here. It reveals, for example, that a number of banks nominally owned by foreigners are ultimately controlled by Russian residents. And vice versa, some banks nominally controlled by Russian-registered entities are in fact foreign-owned. Once a bank is defined as foreign-controlled, we assign it to one of the three categories:

1. SUB — subsidiaries of foreign privately-owned banks; these are banks whose parents are foreign privately-owned banks and whose core business in Russia is commercial banking;
2. GOV — banks controlled by a foreign state-owned bank, foreign government or public sector entity, or an international institution such as the European Bank for Reconstruction and Development;
3. OTH — other foreign-controlled banks; these include:
• banks owned by foreign privately-owned banks but whose core business in Russia is not commercial banking; these are banks engaged in financial markets activity (e.g. investment banking or brokerage), or card and payments processing and settlement;
• banks owned by non-financial institutions, such as foreign automotive companies (BMW, VW, Daimler, Toyota, PSA Peugeot Citroen, Mitsubishi, Renault-Nissan, etc.) or other industrial corporations (e.g. IKEA and Auchan); their core business is the financing of sales in Russia rather than commercial banking in a broad sense;
• banks owned by a foreign investment fund (e.g., Baring Vostok Capital Partners);
• banks owned by foreign individuals.

This assignment to categories is again done on a case-by-case basis and involves judgment. Here we consider the same information as mentioned for state-controlled banks. In addition, we attempt to understand what the core business of the bank is in Russia. To do that we look at the structure of its balance sheet and revenues as well as at the type of licenses the bank possesses. The latter tell us which activities the bank is allowed to engage in. Both pieces of information come from Ref. [9].

Vernikov [12] compares market shares of state- and foreign-controlled banks as defined in this paper with estimates reported in prior literature. He discusses the differences and their likely causes.
